# Sepsis scoring systems and use of the Sepsis six care bundle in maternity hospitals

**DOI:** 10.1186/s12884-021-03921-3

**Published:** 2021-07-23

**Authors:** Nouf Abutheraa, June Grant, Alexander B. Mullen

**Affiliations:** 1 Strathclyde Institute of Pharmacy and Biomedical Science in the University of Strathclyde, Glasgow, UK; 2 Obstetrics & Gynaecology, Women & Children’s Services at the NHS Greater Glasgow & Clyde, Glasgow, UK

**Keywords:** Sepsis, Sepsis scoring systems, Sepsis diagnosis, Obstetric, Maternity, Systemic inflammatory response syndrome, SIRS, qSOFA, MEWS, Early warning systems, Sepsis six care bundle

## Abstract

**Background:**

This study aimed to assess the predictive power of three different Sepsis Scoring Systems (SSSs), namely maternity Systematic Inflammatory Response Syndrome (mSIRS), quick Sepsis-related Organ Failure Assessment (qSOFA) and Modified Early Warning System (MEWS) in identifying sepsis by comparing them with positive culture. This study also sought to evaluate compliance with using the Sepsis Six Care Bundle (SSCB) operated in an individual health board.

**Methods:**

A retrospective cohort study was conducted in 3 maternity hospitals of a single Scottish health board that admitted 2690 pregnancies in a 12 weeks period in 2016. Data for study was obtained from medical notes, handheld and electronic health records for women who were prescribed antibiotics with a confirmed or suspected diagnosis of sepsis. Data on clinical parameters was used to classify women according to mSIRS, qSOFA and MEWS as having sepsis or not and this was compared to results of positive culture to obtain sensitivity, specificity, positive predictive value (PPV), negative predictive value (NPV) and area under Receiver Operating Characteristic curve (AUROC) along with their 95% confidence intervals. Data was also obtained on SSCB compliance.

**Results:**

A total of 89 women were diagnosed with sepsis, of which 14 had missing data, leaving 75 for final analysis. Sensitivity, specificity, PPV, NPV and AUROC of mSIRS and MEWS were almost similar with AUROC of both being around 50%. Only 33 (37.1%) had identifiable sepsis six sticker displayed on medical notes and only 2 (2.2%) had all elements of SSCB delivered within the recommended one-hour post-diagnosis period. Blood culture and full blood count with other lab tests had been performed for most women (97%) followed by intravenous antibiotics and fluids (93.9%).

**Conclusions:**

mSIRS and MEWS were quite similar in detecting sepsis when compared to positive culture, with their ability to detect sepsis being close to chance. This underlines the need for creating a valid SSS with high sensitivity and specificity for clinical use in obstetric settings. Clinical use of SSCB was limited despite it being a health board policy, although there is considerable possibility of improvement following detailed audits and removal of barriers for implementing SSCB.

**Supplementary Information:**

The online version contains supplementary material available at 10.1186/s12884-021-03921-3.

## Background

Sepsis was initially defined at the 1991 ACCP/SCCM Consensus Conference as “a host’s systemic inflammatory response syndrome (SIRS)” [1], based on a score obtained using the patient’s temperature, heart rate (HR), white cell count (WCC) and respiratory rate (RR) [1, 2]. In an obstetric population, changes associated with pregnancy and labour can make the SIRS baseline parameters misleading for the identification of sepsis [3]. These alterations in a woman’s normal physiology can mask the initial phase of sepsis and delay diagnosis, hindering appropriate clinical intervention and subsequent recovery [4]. Interpretation of other biomarkers of infection such as C-reactive protein (CRP) and WCC can also vary with the mode of delivery that women experience [5, 6]. As a consequence, a number of alternate Sepsis Scoring Systems (SSSs) were developed for maternity use such Sepsis in Obstetric Score (SOS) [3] or others such as maternity SIRS (mSIRS) [7] which are in use only in certain medical systems.

Not only for obstetric cases but even for the normal population, a re-evaluation of the definition of sepsis and SIRS after 24 years of clinical use found it to be deficient in both specificity and sensitivity, as it failed to capture many true cases of sepsis and identified infections that are not necessarily sepsis [1]. The re-evaluation indicated that the SIRS construct, although useful in identifying patients with infection, had limited specific applicability to sepsis [1]. The definition of sepsis was overhauled during the 3rd International Consensus Definition for Sepsis and Septic Shock [1]; the new definition of sepsis being “life-threatening organ dysfunction caused by a dysregulated host response to an infection” [1]. In order to detect sepsis in line with the new definition, a Sepsis-related Organ Failure Assessment (SOFA), and a quick SOFA (or qSOFA) were created, comprising only three indices: mental status, systolic blood pressure (SBP) and RR [1]; although SOFA or qSOFA may not capable of replacing SIRS [2].

Apart from variations of SIRS, SOFA and qSOFA, the Royal College of Obstetricians and Gynaecologists UK, endorsed a pregnancy-specific scoring system, the Modified Obstetric Early Warning Score (MOEWS) [3, 8] which was related to Modified Early Warning Score (MEWS) for non-obstetric population [9]. A number of MOEWS are in use in various facilities globally but very few have been validated [9]. Evidence suggests that most of these MOEWS are not as good when compared to MEWS for obstetric use [9].

In addition to developing various SSSs for early detection of sepsis during obstetric care, it was also essential to recognize that patients’ deterioration can be limited by the early management of sepsis. This has led to the application of care bundles as a mechanism for minimizing harm and enhancing patient care [10]. The Sepsis Six Care Bundle (SSCB) was recently introduced into the maternity hospitals of the health region under examination in this study, in the form of a sticker on patients’ notes, with the aim of delivering all six elements of the bundle (Additional file 1: Sepsis Six Sticker) within 1 h of sepsis being provisionally diagnosed. However, compliance has not been uniform and in line with the suggestions of SSCB [11].

Thus, there are many variations of SSSs in obstetric use across the world and very few have been validated including some we were interested in. Hence, we assessed mSIRS (since this was recommended by the health board where this study was conducted), qSOFA (an improvement over the earlier SIRS) and MEWS (which performed better than all MOEWS [9]) in identifying sepsis by applying these criteria to all pregnant women treated for sepsis and by comparing them to positive culture as a gold standard. In addition, we also evaluated compliance with using the SSCB.

## Methods

### Study design and settings

A retrospective observational cohort study was conducted within three maternity hospitals situated in a single Scottish health region that admitted 2690 pregnant women in a 12 weeks period in 2016. The study sample comprised all women admitted to these maternity hospitals during the study period who received antibiotic therapy for a suspected or confirmed diagnosis of sepsis.

### Data collection and subject identification

Patients were identified through hospital handover sheets or/and drug Kardexes® (records of patients’ medication prescription and administration). Women ≤16 years of age were excluded. Women were also excluded if antibiotics were prescribed prophylactically; such women were included only if they required additional treatment following a diagnosis of sepsis.

Individual demographic characteristics including patient’s age, body mass index (BMI), gestational age, mode of delivery and unit of hospitalization were collected. Data on each patient’s temperature, heart rate (HR), respiratory rate (RR), systolic blood pressure (SBP) and mental status were collected from individual MOEWS charts, while individual data on WCC and microbiology reports (positive culture) were collected from patient electronic health records (EHRs).

Patient’s clinical notes were then reviewed to determine whether the SSCB had been commenced and used appropriately. This evaluation was not reported for patients who were commenced on the bundle without the use of the SSCB sticker.

### Data management

Based on the various SSSs that were evaluated, the values of all required patient parameters such as HR, RR, SBP, mental status etc. were dichotomized to be normal or abnormal. These dichotomized values were then combined to create a composite score for a particular SSS to evaluate whether a patient had sepsis or not based on a particular SSS. Positive culture results were used to evaluate the selected SSSs.

### Data analysis

All analysis was performed using SPSS Statistics for Windows, Version 23.0 (Armonk, NY: IBM Corp.). Means, standard deviations and range was calculated for all continuous variables. Counts and percentages were calculated for all categorical variables. We evaluated three SSSs – mSIRS, qSOFA and MEWS (we selected MEWS and not any of the MOEWS since a recent study discovered MOEWS to be less effective than MEWS in detecting sepsis early in obstetric settings [9]) with positive culture results as gold standard to obtain sensitivity, specificity, positive predictive value (PPV) and negative predictive value (NPV) along with their 95% confidence intervals (CIs). To summarize our evaluation of SSSs we calculated Receiver Operating Characteristic (ROC) curve along with area under the ROC (AUROC) and 95% CIs. We also assessed compliance with the SSCB in our study by calculating the counts and percentages of patients receiving the six elements of SSCB.

## Results

This study included a total of 89 (3.3%) women, diagnosed with sepsis, from a total of 2690 pregnancies, in a single health region in Scotland during a 12-week study period in 2016.

Table 1 summarises the demographic details of the 89 women based on the data available in the EHRs. The average age of these women was 29.8 (± 5.3) years and ranged from 19 to 45 years. A total of 19 (24.7%) women were classified as obese with Body Mass Index (BMI) equal to or greater than 30, with most women (46.7%) having a normal BMI. Most women (94%) were in the third trimester, with the greatest proportion of deliveries occurring via emergency caesarean section (46.1%), followed by spontaneous vaginal delivery (24.7%). The majority of women (89.9%) were admitted either into the antenatal or postnatal wards with very few in high dependency unit (7.9%) or intensive care unit (2.2%).

**Table 1:** Demographic information of pregnant women (*N* = 89) diagnosed with suspected sepsis in maternity wards of 3 hospitals of a health board in Scotland

Demographic variables	Values (*N* = 89)
Age	
Mean ± SD (Min-Max)	29.8 ± 5.3 (19–45)
BMI (kg/m^2^)^a^	
Normal (18.5–24.9)	36 (46.7%)
Overweight (25–30)	22 (28.6%)
Obese (≥ 30)	19 (24.7%)
Gestational age^b^	
First trimester	0
Second trimester	5 (6%)
Third trimester	79 (94%)
Delivery mode^c^ – n (%)	
Emergency caesarean section	41 (46.1%)
Spontaneous vaginal delivery	22 (24.7)
Instrumental delivery	14 (15.7%)
Eelective caesarean section	1 (1.1%)
Unit of hospital admission – n (%)	
Antenatal or Postnatal ward	80 (89.9)
High Dependency Unit	7 (7.9)
Intensive Care Unit	2 (2.2)

^a^Data were missing for 12 women; ^b^Data were missing for 5 women; ^c^Data were missing for 11 women, of whom 7 were diagnosed with sepsis in the antenatal period

Data for evaluating Sepsis Scoring Systems (SSSs) was available only for 75 out of 89 (84.3%) women. Various Sepsis Scoring Systems, namely mSIRS, qSOFA and MEWS (Table 2), were compared with positive culture (Table 3), to obtain characteristics such as sensitivity, specificity, PPV, NPV and AUROC. mSIRS and MEWS were found to be comparable with regards to most characteristics – sensitivity [mSIRS: 0.655 (95% CI – 0.643-0.668); MEWS: 0.690 (95% CI – 0.678-0.702)], specificity [mSIRS: 0.391 (95% CI – 0.379-0.404); MEWS: 0.304 (95% CI – 0.292-0.316)], PPV [mSIRS: 0.404 (95% CI – 0.391-0.417); MEWS: 0.385 (95% CI – 0.372-0.397)], and NPV [mSIRS: 0.643 (95% CI – 0.630-0.655); MEWS: 0.609 (95% CI – 0.596-0.621)]. AUROC for both were close to 50% (mSIRS AUROC – 0.524 (95% CI - 0.388-0.659) and MEWS AUROC – 0.497 (95% CI - 0.354-0.639). Thus, specificity, PPV, NPV and AUROC were slightly higher for mSIRS compared to MEWS. qSOFA on the other hand was positive (score ≥ 2) for only 2 women both of whom had negative cultures (Table 3).

**Table 2:** Comparison chart of Sepsis Scoring Systems useful for maternity care for pregnant women diagnosed with probable sepsis in maternity wards of 3 hospitals of a health board in Scotland

Parameters or Criteria used in Sepsis Scoring Systems	Sepsis Scoring Systems Analysed in the Current Study^a^			
	SIRS^a^	Maternity SIRS	qSOFA	MEWS
Temperature (°C)	< 36 or > 38	< 36 or > 38	–	≤ 36 or > 38
Heart rate (bpm)	> 90	> 100	–	≤ 50 or > 110
White cell count	< 4 or > 12 × 10^9^/L	< 4 or > 16 × 10^9^/L	–	
Respiratory rate (bpm)	> 20	> 20	≥ 22	< 9 or > 14
Systolic blood pressure (mmHg)	–	< 90	≤ 100	≤ 100 or > 199
Mental status	–	Altered mental status	Altered mental status	Altered mental status

^a^All except SIRS were compared in the study. SIRS has been made a part of the table to show its differences with Maternity SIRS

**Table 3:** Comparison of Sepsis Scoring Systems against Positive Culture^a^ for pregnant women (*N* = 75) diagnosed with probable sepsis in maternity wards of 3 hospitals of a health board in Scotland

Characteristics	Sepsis Scoring Systems for Maternity Care % (95%CI)^b^		
	Maternity SIRS	qSOFA^c^	MEWS
Sensitivity	0.655 (0.643–0.668)	0	0.690 (0.678–0.702)
Specificity	0.391 (0.379–0.404)	0.957 (0.951–0.962)	0.304 (0.292–0.316)
PPV	0.404 (0.391–0.417)	0	0.385 (0.372–0.397)
NPV	0.643 (0.630–0.655)	0.603 (0.589–0.603)	0.609 (0.596–0.621)
AUROC	0.524 (0.388–0.659)	0.301 (0.045–0.558)	0.497 (0.354–0.639)

^a^29 patients had a positive culture; ^b^ *CI* Confidence Interval; ^c^Only 2 patients had qSOFA score of 2 and both had negative cultures

We also evaluated the use of Sepsis Six Care Bundle (SSCB) for 33 (37.1%) women (Table 4), who had the sepsis six sticker on their medical notes, and discovered that most women received 5 of the 6 elements of SSCB. Most women had blood culture, and full blood count with other lab tests performed (97%) followed by intravenous antibiotics and intravenous fluids (93.9%). Catheterization was done for 78.8% women and 27.3% women administered oxygen. It is important to note here that only 2 cases received all 6 elements of SSCB within the prescribed 1 h of being diagnosed with sepsis – at 35 and 40 min from time zero.

**Table 4:** Details of compliance with each element of the Sepsis Six Care Bundle (SSCB) for pregnant women (*N* = 33) diagnosed with probable sepsis in maternity wards of 3 hospitals of a health board in Scotland

Care/therapy	Compliance with the care at any time point n (%)
Oxygen	9 (27.3%)
Blood culture	32 (97%)
Full blood count and other lab tests	32 (97%)
Intravenous antibiotics	31 (93.9%)
Intravenous fluid	31 (93.9%)
Catheter	26 (78.8%)

Note: Only for 2 cases SSCB was delivered within the prescribed one-hour period - reported times being 35 and 40 min from time zero

## Discussion

Our study on 89 women, in a single health board in Scotland, which compared three different SSSs – namely mSIRS, qSOFA and MEWS, with positive culture, revealed comparable performance by mSIRS and MEWS with similar values for sensitivity, specificity, PPV, NPV and AUROC. Most importantly the values for AUROC for both mSIRS and MEWS were around 50% indicating that their ability to predict sepsis was close to chance compared to positive culture. Estimates for qSOFA were unreliable in our study due to low number of women with positive qSOFA.

On evaluating compliance with the use of SSCB, only 33 (37.1%) of these women were found to have sepsis six sticker on their medical notes denoting low levels of compliance with providing timely recommended sepsis care. Among those who received SSCB, only 2 women received it within the recommended 1 h following diagnosis. While most received 4/6 elements of SSCB, only around 79% received catheterization and the minority (27.3%) were administered oxygen.

Our findings regarding comparison of SSSs with positive culture threw up meaningful results only for mSIRS and MEWS. mSIRS is a modification of the SIRS criteria and has been in use in the hospitals of the health board since 2015 [7]. mSIRS performance was similar to that of MEWS which indicated that mSIRS was probably one of the better performing SSS in obstetric settings. This can be inferred in the light of findings where MEWS was compared to six different published MOEWS and was found to perform better than all of them [9]. The different MOEWS were reported to have low PPV, ranging between 1.4 and 5.1%, while AUROC ranged from 0.52 to 0.72 [9].

The use of SIRS criteria in the original definition of sepsis were not specific and its values can be elevated in cases of other non-infectious disease such as burns or injury and in cases unrelated to infection [12, 13]. In addition, women undergo changes in their physiological and laboratory measurements during pregnancy and post-partum periods. These changes overlapped with the SIRS criteria and made the diagnosis critical, particularly when respiratory rate, heart rate, white cell count and blood pressure values, expected to be outside the adult normal reference range [12, 13].

An important thing to note here is the continued use of mSIRS (though SIRS was found highly inadequate after decades of use [1]) in the health board that was studied and the presence of numerous MOEWS in various obstetric setting across the world; this points towards the lack of consensus [13] about a single validated SSS which is needed for use in obstetric settings and is currently missing.

It has been proposed that the qSOFA definition should be able to identify women at an early stage of severe maternal infection to allow healthcare practitioners to initiate treatment [14]. The definition of severe maternal sepsis excludes the early stage of sepsis and delays the initiation of treatment for these women. The new definition of maternal sepsis is “a life-threatening condition defined as organ dysfunction resulting from infection during pregnancy, childbirth, post-abortion, or the postpartum period” [15]. When applied to our sample, this criterion was able to determine sepsis in only two women. It seems that it can be used in very sick women to determine their need for ICU admission, but has limited applicability to sepsis cases in maternity wards.

Finally, we considered comparing the various SSSs to positive culture. Our results with different SSSs show uncertainty about systems to be used in diagnosis and culture-proven sepsis is still the diagnostic gold standard. There were only 29 positive culture results among 75 women (38.6%), which is in line with the evidence [16]. Despite being the gold standard there are severe deficiencies in using culture tests in clinical settings due to poor specificity and precious time lost in waiting for culture results [16]. These shortcomings with culture tests further underline the need for creating an effective SSS with high sensitivity and specificity for early use in clinical settings.

The early identification and management of sepsis in maternity is recommended [17], as delay in diagnosis or treatment can lead to maternal mortality and morbidity, which are associated with a delay in administering the appropriate therapy and management to these women [18]. Culture-proven sepsis results were obtained 48 h after therapy had been initiated for these women, and an early identification tool is necessary so that treatment can be delivered early. The limited applicability of various biomarkers due to the non-specific or non-sensitive nature of these criteria, arising from the altered physiological functions of pregnant women, could lead to under- or over-treatment. The unnecessary use of antibiotic therapy to treat maternal sepsis could drive antibiotic resistance and further medical complications [4, 19], while delay in managing sepsis can result in many complications including organ failure, hysterectomy and death [18–20].

The use of clinical judgement is currently recommended to facilitate the diagnosis of sepsis, which should not rely solely on SSSs or laboratory tests [20]. Chorioamnionitis cases, for example, may have a clinical diagnosis and a histological diagnosis but women might not have a positive microbiological culture while being treated with antibiotic for clinical chorioamnionitis [20]. Overall, the current system of diagnosing sepsis hardly relies on the effectiveness of the early warning scores and this might be a hindrance for validating these tools in identifying ill patients.

In order to make SSSs more effective, addition of biomarkers has been suggested. Procalcitonin (PCT) shows better prediction of sepsis compared to CRP; nevertheless, there is a reported lack of accuracy [21]. Serum PCT rises and peaks in a short time compared to CRP and it has been found to be useful in modern clinical practice. However, there is limited information about PCT in pregnancy, and determining the most appropriate reference range of this biomarker in pregnant women [22]. Its high level of negative predicted values leads to a misleading identification of sepsis [23]. Martín and colleagues recommend the design of a scoring system based on combined biomarker values including CRP, PCT and lactate to aid physicians in their treatment decisions, which has the potential to reduce the use of antibiotics and of culture tests [24].

With respect to compliance with SSCB, recent studies have indicated that very few patients received all the six interventions of the bundle within the recommended 1 h and the elements were not consistently applied [11]. This is in line with our findings. There are possibly a number of barriers that might hinder the timely and effective delivery of SSCB – a few have been described such as insufficient audit and feedback, poor teamwork and communication, inadequate training, lack of resources and concerns about using SSCB in certain patients [25]. However, health personnel also displayed positive factors in favour of SSCB such as confidence in knowledge and skills, and belief in the benefits of SSCB among others [25]. Since compliance with SSCB can vary among facilities, regular audits, continued use, further research into non-compliance at facility level and improving the strengths of the staff at facility level can lead to better implementation of SSCB.

## Conclusions

In conclusion, access to a large database from maternity hospitals of a health board provided appreciable data on pregnant women with sepsis. Overall, the study indicated that mSIRS and MEWS were quite similar and weak in their characteristics in detecting sepsis in obstetric settings when compared to positive culture as a gold standard. Also, compliance with SSCB was low and administering care for all 6 elements in SSCB was not uniform. This study highlights the need for a rapid, point of care technology, incorporating a panel biomarker of greater specificity/sensitivity to support a SSS with superior prediction powers than currently available tools.

## Supplementary information

**Additional file 1:** Sepsis Six Sticker.

## Data Availability

The datasets used and analysed during the current study are available from the corresponding author on reasonable request.

## Abbreviations

AUROC: Area under the receiver operating characteristic curve
BMI: Body mass index
CRP: C-reactive protein
HDU: High dependency unit
HR: Heart rate
ICU: Intensive care unit
MEWS: Modified early warning score
MOEWS: Modified obstetric early warning score
mSIRS: Maternity systemic inflammatory response syndrome
NPV: Negative predicted value
PCT: Procalcitonin
PPV: Positive predicted value
qSOFA: Quick sepsis-related organ failure assessment
ROC: Receiver operating characteristic
RR: Respiratory rate
SBP: Systolic blood pressure
SIRS: Systemic inflammatory response syndrome
SSCB: Sepsis six care bundle
WCC: White cell count

## References

[1] Singer M, Deutschman CS, Seymour CW, Shankar-Hari M, Annane D, Bauer M, Bellomo R, Bernard GR, Chiche JD, Coopersmith CM, Hotchkiss RS, Levy MM, Marshall JC, Martin GS, Opal SM, Rubenfeld GD, van der Poll T, Vincent JL, Angus DC (2016). The third international consensus definitions for Sepsis and septic shock (Sepsis-3). JAMA..

[2] Vincent J-L, Martin GS, Levy MM (2016). qSOFA does not replace SIRS in the definition of sepsis. Crit Care.

[3] Albright CM, Ali TN, Lopes V, Rouse DJ, Anderson BL (2014). The Sepsis in Obstetrics Score: a model to identify risk of morbidity from sepsis in pregnancy. Am J Obstet Gynecol.

[4] Cordioli RL, Cordioli E, Negrini R, Silva E (2013). Sepsis and pregnancy: do we know how to treat this situation?. Rev Bras Ter Intensiva.

[5] Keski-Nisula L, Kirkinen P, Ollikainen M, Saarikoski S (1997). C-reactive protein in uncomplicated parturients delivered by cesarean section. Acta Obstet Gynecol Scand.

[6] Arbib N, Aviram A, Gabbay Ben-Ziv R, Sneh O, Yogev Y, Hadar E (2016). The effect of labor and delivery on white blood cell count. J Matern-Fetal Neonatal Med.

[7] NHS Greater Glasgow & Clyde. Obstetric guidelines; maternal sepsis. 2015;1–9.

[8] Royal College of Obstetricians and Gynaecologists. Bacterial Sepsis in Pregnancy. Published 2012. https://www.rcog.org.uk/globalassets/documents/guidelines/gtg_64a.pdf Accessed 03 June 2020.

[9] Edwards SE, Grobman WA, Lappen JR, Winter C, Fox R, Lenguerrand E (2015). Modified obstetric early warning scoring systems (MOEWS): validating the diagnostic performance for severe sepsis in women with chorioamnionitis. Am J Obstet Gynecol.

[10] Gilbert JA (2018). Sepsis care bundles: a work in progress. Lancet Respir Med.

[11] Frankling C, Patel J, Sharif B, Melody T, Yeung J, Gao F, Szakmany T (2019). A snapshot of compliance with the sepsis six care bundle in two acute hospitals in the west midlands, UK. Ind J Crit Care Med.

[12] Joynes E (2016). More challenges around sepsis: definitions and diagnosis. J Thorac Dis.

[13] Turner MJ (2019). Maternal sepsis is an evolving challenge. Int J Gynecol Obstet.

[14] Bonet M, Nogueira Pileggi V, Rijken MJ, Coomarasamy A, Lissauer D, Souza JP, Gülmezoglu AM (2017). Towards a consensus definition of maternal sepsis: results of a systematic review and expert consultation. Reprod Health.

[15] World Health Organization. Statement on maternal sepsis. [Internet]. 2017. Available from: https://www.who.int/reproductivehealth/publications/maternal_perinatal_health/maternalsepsis-statement/en/ Accessed 16 October 2020.

[16] Greer O, Shah NM, Johnson MR (2020). Maternal sepsis update: current management and controversies. Obstet Gynaecol.

[17] Shankar-Hari M, Phillips GS, Levy ML, Seymour CW, Liu VX, Deutschman CS, Angus DC, Rubenfeld GD, Singer M, for the Sepsis Definitions Task Force (2016). Developing a new definition and assessing new clinical criteria for septic shock: for the third international consensus definitions for Sepsis and septic shock (Sepsis-3). JAMA..

[18] Bauer ME, Lorenz RP, Bauer ST, Rao K, Anderson FWJ (2015). Maternal deaths due to Sepsis in the state of Michigan, 1999-2006. Obstet Gynecol.

[19] Llor C, Bjerrum L (2014). Antimicrobial resistance: risk associated with antibiotic overuse and initiatives to reduce the problem. Ther Adv Drug Saf.

[20] Wood A, Post A, Swamy G, Murtha A, Heine RP, Grotegut C (2016). 13: obstetric complications associated with sepsis. Am J Obstet Gynecol.

[21] Mangogna A, Agostinis C, Ricci G, Romano F, Bulla R (2019). Overview of procalcitonin in pregnancy and in pre-eclampsia. Clin Exp Immunol.

[22] Kibe S, Adams K, Barlow G (2011). Diagnostic and prognostic biomarkers of sepsis in critical care. J Antimicrob Chemother.

[23] Faix JD (2013). Biomarkers of sepsis. Crit Rev Clin Lab Sci.

[24] National Institute for Health and Care Excellence. Procalcitonin testing for diagnosing and monitoring sepsis (ADVIA Centaur BRAHMS PCT assay, BRAHMS PCT Sensitive Kryptor assay, Elecsys BRAHMS PCT assay, LIAISON BRAHMS PCT assay and VIDAS BRAHMS PCT assay). Published 2015. https://www.nice.org.uk/guidance/dg18/ Accessed 03 June 2020.

[25] Roberts N, Hooper G, Lorencatto F, Storr W, Spivey M (2017). Barriers and facilitators towards implementing the Sepsis Six care bundle (BLISS-1): a mixed methods investigation using the theoretical domains framework. Scand J Trauma Resusc Emerg Med.

