# Supplementary figures and images for: Sepsis scoring systems and use of the Sepsis six care bundle in maternity hospitals
Source: BMC Pregnancy Childbirth. 2021 Jul 23;21:524. doi: 10.1186/s12884-021-03921-3 (PMC8305522; doi:10.1186/s12884-021-03921-3)

**Appendix S1: Sepsis Six Sticker**


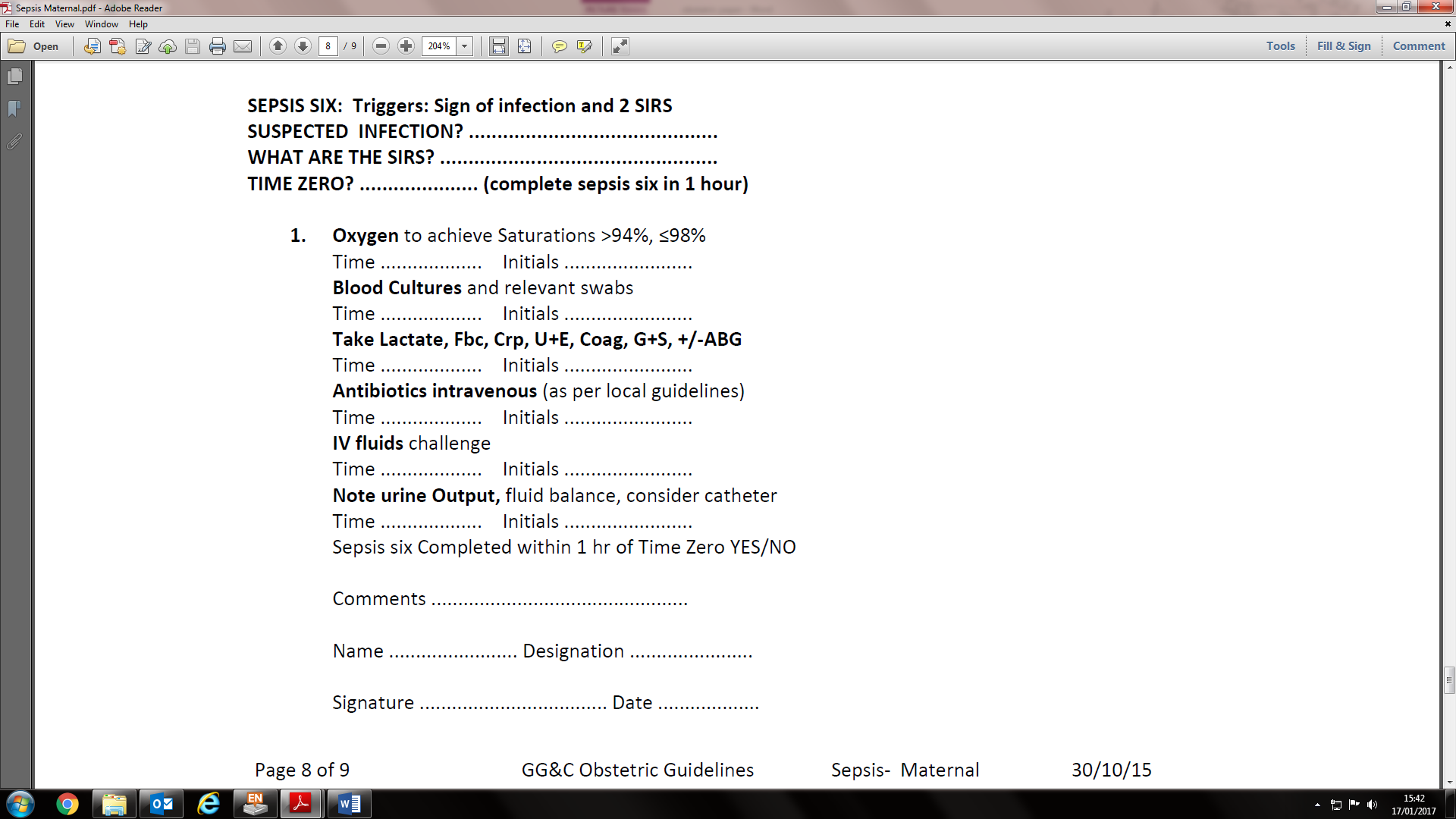

Supplement: Supplementary file 1 — Additional file 1: Sepsis Six Sticker. [file 12884_2021_3921_MOESM1_ESM.docx]
